# Salient features of the National Program for Control of Blindness during the XI^th^ five-year plan period

**DOI:** 10.4103/0301-4738.55064

**Published:** 2009

**Authors:** R Jose, A S Rathore, V Rajshekar, Sandeep Sachdeva

**Affiliations:** Directorate General of Health Services, Ministry of Health and Family Welfare, Government of India, Nirman Bhawan, New Delhi-11, India. E-mail: addldghs-mohfw@nic.in

The national five-year planning process starts with consultation with various stakeholders including central and State governments/Union territories (UTs), experts in the field of ophthalmology and public health, professional bodies, All India Ophthalmological Society (AIOS), national level institutes andrepresentatives of voluntary organizations/non-government organizations (NGOs) by holding workshops, deliberations and meetings. The plan along with technical and financial justification in the appropriate format goes through the process of review and regulatory clearance from various levels in the ministry of health and family welfare, finance ministry, planning commission, expenditure finance committee and only after cabinet approval does the plan get implemented. The Cabinet Committee on Economic Affairs [CCEA], the highest body constituted by the Parliament of India, has endorsed and approved a budget of INR 1250 [twelve hundred and fifty] crores on 3 October 2008 for the XI^th^ five-year (2007-12) plan period under the National Program for Control of Blindness [NPCB]. This editorial brings out the salient features of the approved plan.

Keeping in view the strength and weakness of the program, the XI^th^ five-year plan envisages continuation of centrally sponsored scheme with some modification and introduction of new schemes. The challenges and issues that are being addressed with renewed emphasis are diabetic retinopathy, glaucoma, childhood blindness like congenital cataract, squint, amblyopia, strabismus, besides the core diseases like cataract, focal trachoma, refractive errors and low vision. A decision has been taken with regard to integration of NPCB with the National Rural Health mission [NRHM] for ensuring optimal utilization of the existing infrastructure at various levels of the healthcare system including fund flow to States and UTs.

Financial assistance[1] in the form of Grant-In-Aid [GIA] is being provided to the tune of Rs. 60 lakhs for regional institutes of ophthalmology (RIO), Rs. 40 lakhs for medical colleges, Rs. 20 lakhs for district hospitals, Rs. 05 lakhs for sub-district hospitals and Rs. 50 thousand for vision centers. This will strengthen the appropriate institutions with instruments, equipments, consumables, teaching and training aids, furniture and fixtures, in addition to recurring expenditure of state and district health societies. RIO and selected medical colleges are being strengthened to develop into centers of pediatric eye care units, low vision units, and/or retina units. Eye banking activities are being up-scaled through financial assistance up to Rs. 15 lakhs and Rs. 01 lakh for developing eye banks and eye donation centers respectively.

NGOs play a critical role in delivering eye care services in the country and various financial schemes are available through public private partnership mode. Non-recurring one-time financial assistance up to Rs. 30 thirty lakhs on a 1:1 basis is being extended to NGOs for providing free eye care services, especially in underserved/rural areas. Provision of GIA for cataract surgery (up to a maximum of Rs. 750) and other eyediseases (Rs. 1000/- per case) like diabetic retinopathy, glaucoma, childhood blindness and corneal transplantation has been initiated for the first time and the scheme is being made available from this financial (2008-09) year itself. Cataract surgery is performed under daycare but for the patients residing in far-flung/hilly terrain/difficult areas with poor transportation services there is a need for adequate in-patient services. As a result, Rs 75 lakhs have been earmarked for construction of dedicated eye wards, eye operation theaters and mobile ophthalmology vans in northeastern states/hilly/other underdeveloped states. Staff crunch was noticed in most of the states in effective delivery of eye care services hence it has been proposed to appoint additional eye surgeons (250 in number), ophthalmic assistants (425 in number), eye donation counselor (150 in number) on a contractual basis in newly carved out districts and vision centers where there are none.

Financial assistance up to a maximum of Rs. 70,000 per trainee is being provided to 27 eye care institutes in voluntary/NGO sector under the program for enhancing the capacity of public eye surgeons in the sub-specialty of ophthalmology. States/UTs are provided a lump sum amount by the Government of India for refresher training of medical officers, ophthalmic assistant, school teachers and other healthcare staff in eye care services. Community link worker, namely Accredited Social Health Activist (ASHA) and Anganwadi workers have been roped into the program for increasing community awareness. Another initiative that has been taken with regard to improving eye collection in the country is advocacy, informing and sensitizing health personnel via small movie clips on the procedure of eye removal through in-service training institutes in the country. A pilot project of tele-ophthalmology is being implemented at selected sites and states in the country to assess cost-effectiveness, feasibility and acceptability of newer technology. The impact and outcome is to be studied over time. To conclude, the Government of India has directed its efforts towards achieving the goal of elimination of avoidable blindness in the country by the year 2020 and hopefully that will become a reality.

